# Encountering deception in virtual spaces: guidelines for virtual ethnography

**DOI:** 10.3389/fsoc.2023.1163560

**Published:** 2023-09-28

**Authors:** Lisa Lucile Owens

**Affiliations:** University of Massachusetts School of Law, Dartmouth, MA, United States

**Keywords:** deception, virtual, ethnography, research methodology, data transparency

## Abstract

This Perspective Essay draws from an experience of deception in virtual fieldwork and considers implications for those designing methodologies for virtual ethnographies. As qualitative field work increasingly takes place within virtual spaces and through virtual means, researchers are faced with critical dilemmas in the processes of data gathering and verification. One of these dilemmas concerns ensuring data validity and facticity if encountered with research subjects who are deceptive about their identity, experiences, or relationship to the field of research. This Perspective Essay offers specific guidelines concerning articulating the nature and possibilities of deception in virtual spaces, identifying deceptive data, and what to do with deceptive data in order to maintain data validity and transparency.

## Introduction

Entering the realm of virtual research during the widespread social distancing measures and shutdowns related to the Covid-19 pandemic beginning in 2020, I and many others began the process of adapting research and methodologies to primarily virtual contexts. During that time, I encountered a woman named Colleen^1^ who I was able to ascertain was taking on a deceptive identity in order to participate in research studies. This Essay builds on a previous Article written about my experience with Colleen by exploring some of the methodological implications of intentional deception for ethnography conducted in virtual spaces. In writing it, the intended audience are first time ethnographers navigating virtual spaces. Accordingly, I provide a series of guidelines for each stage of the research process, including the research design phase, the data gathering and analysis phase, and the reporting phase.

Virtual methodologies present many of the same challenges and concerns as in-person methods; however, virtual methods also introduce certain new challenges or change the nature of old ones (Owens, 2022). Although virtual and physical spaces can often seem to be analogous, virtual spaces can both shield and reveal in ways that physical spaces do not. Some of the features of virtual space may make it more vulnerable to deception. For example, the liminal spaces, points of transition and waiting, of virtual spaces are different than in physical spaces. Many of the physical spaces where observations take place are shared in-person, but not virtually, changing the research experience (See, e.g., Gorski, 2021). These moments and spaces are one way in which the researcher orients themselves, gauging the authenticity and complexity of those whom they are interacting with. Additionally, with virtual space, the barriers to entry are lower for those being observed, perhaps meaning that there is a greater risk of deception in virtual space.

Virtual research presents many advantages as well. For ethnography, without the burden of travel, more observations with more research subjects may be conducted in a shorter amount of time. For ethnographic interviews, scheduling may be easier if research participants do not need to commit to traveling physically to an interview location. A wide range of groups are accessible to a wide range of researchers in comparison to in-person ethnography, and the possibility of multi-sited, global ethnography is made real. Without the burden of travel, more observations with a single research participant may also be easier to conduct, making ethnographic methodologies more accessible for researchers and participants. As well, encountering the inner lives and spaces of research participants through virtual communication methods can add to the depth of data.

The possibility of deception is particularly salient in terms of the validity, accuracy and transparency of online research. While deception can take many forms, for the purposes of this paper I focus on deception that is intentional on behalf of the research subject. Intentional deception in virtual spaces can take many forms. For example, it can involve deception about one’s relationship to the research, as was the case with Colleen. It can involve deception about one’s own identity in online spaces. And it can involve deception or embellishment about one’s story or experiences. This Essay primarily provides some practical methodological guidelines around intentional deception in virtual ethnography.

### Encountering colleen

I’ve documented a more expansive description of how I negotiated my relationship with Colleen in a preceding article (see Footnote 1). Here, I summarize this relationship for the purposes of giving context to the rest of this Essay. I first encountered Colleen in an email when she responded to a Craigslist ad recruiting residents of New York City public housing for an interview-based research study. Initially, I experienced Colleen as an ideal subject. She communicated clearly and was verbose in her responses. For example, whereas most respondents answered my initial written questions as briefly as possible (“yes” or “no”), Colleen gave detailed answers. However, it was only a few minutes into our initial synchronous verbal conversation that I began to suspect that Colleen did not have the experience that she claimed and was not likely to have ever been a resident of New York City public housing. When asked questions in ‘real time’, Colleen could not adequately fabricate answers to questions like “What do your neighbors do for a living?” (Answer: “Snitches get stitches.”) or “What is your neighborhood [reportedly midtown Manhattan] like at night?” (Answer: “Very scary”). Her answers were sufficiently implausible that I politely ended the interview quite early on, paying her the promised incentive fee but marking her data as likely deceptive and separating it from other data. At this time, I strongly suspected that Colleen was being deceptive, but I did not yet have definitive ‘proof’ or admission of deception. I was also unsure about what to do with Colleen’s data and what my responsibility was in reporting it to future readers of related work.

The second time I encountered Colleen was in her answer to an advertisement attempting to recruit preliminary interviewees for a second, unrelated research study. In her response, Colleen used the same unique ‘real’ (legal) name, and I was immediately able to flag the email. This time, the data she gave was fundamentally at odds with the data that she had previously given. As I now had confirmation that Colleen was engaging in deception, I was emboldened to try to engage her in a conversation about how and why she was doing what she was doing. I wrote her back with a direct request to speak to her about how she went about, in my words, “posing as someone who meets criteria for these [research] studies.” She also indicated that she was happy to speak with me about her “story.”

Colleen described herself as a people pleaser who was able to intuit what researchers wanted to hear in interviews. She felt that she had been very successful earning an income through participation in research studies that paid incentive fees and were completed online so that she did not need to travel. At the time we spoke, she reported that I had been the only researcher to ‘figure it out’ (though I suspect others did, perhaps just not confronting her) and that she had participated in ‘hundreds’ of research studies. Colleen did not change every aspect of her persona for the research studies, only the relevant ones. For example, Colleen did not have a husband at the time that we spoke, though she indicated that she did, describing him as “the sexiest Puerto Rican guy” and basing his characteristics on a celebrity that she admired. When I asked her about how she met her husband in the initial interview, she was able to give a detailed, plausible, and gushingly romantic story. I would have had no reason to question this part of her interview, should it have existed in a vacuum.

However, Colleen was less able to answer specific questions that focused on less universal subject matter, instead relying on cultural tropes in those circumstances that did not resonate as authentic. Over email, for the site and parameter specific answers to the questions I had asked initially, Colleen had had the time to carefully consider her answers. For example, she had conducted research via the internet to find the specific name of a public housing complex; however, in the midst of a real-time, synchronous conversational exchange, she did not have time to conduct this outside research.

In our conversation, Colleen indicated that when selecting studies to participate in, she sought to avoid research studies that involved complex knowledge, triangulation of data, or detailed storytelling about subjects she did not have personal knowledge, such as medical conditions. Instead, Colleen sought to participate in studies involving more universal experiences which were also highly individualized (i.e., relationships or work), or experiences which could not easily be triangulated or disproven. For example, in order to provide a plausible rendition of experience with a specific medical condition, one would need to become intimately familiar with the symptoms, treatments and communities of a condition. However, a rendition of an experience working is an almost universalizable experience which is, at the same time, highly individualized and difficult to confirm.

Colleen’s success deceiving virtual researchers has methodological implications. Colleen was encountered in the context of virtual interviews, and considerations about incentive fees, recruitment, interview structure and design are immediately brought to bear. However, other virtual methodologies are also implicated and the following section will consider some of those implications for virtual ethnography.

### [A few] guidelines for virtual ethnography

Colleen highlights the fact that virtual spaces do facilitate those who wish to deceive in ways that physical spaces do not, and that ethnographers operating in virtual spaces should expect to encounter deception. Below, I discuss three sets of guidelines for researchers entering into ethnographic study of virtual spaces which emerge from my own experience in virtual fields of research.

#### The research design phase: articulating the nature and possibilities of deception in virtual spaces

The performance of social life in online spaces may be easier to creatively maintain or manipulate, which makes understanding deception in virtual spaces complex. Various forms of anonymity in virtual space, including around data privacy, also complicates the concept of deception. Whereas data verification and “questioning and corroborating” data is generally a part of ethnographic research (Murphy et al., 2021, p. 50), virtual spaces, perhaps more easily than physical spaces, allow individuals to obscure, invent, enhance and borrow identities, experiences and modes of interaction. The motivations for this vary and include fears about personal safety or risk, and, at times, may also represent attempts at self-invention or experimentation. Further complicating this, participant anonymity or self-enhancement in virtual space is sometimes socially normative or considered best practices.

In Sociologist Forberg’s (2021) virtual ethnographic work on extremist conspiracy theorists, part of his data gathering required ‘lurking’ anonymously in virtual message board spaces. There, anonymity or semi-anonymity was a common practice as a risk management strategy to prevent doxxing. As well, in the community he was observing there was also a running suspicion about infiltration of the space by ‘fake’ community members (2021). Contrasting further, in Sociologist Julia Goldman-Hasbun’s study of hate speech in virtual message boards, total anonymity was the norm and basis for the kind of expression the researcher was studying (2022). On a totally anonymized message board or in a forum where anonymity is common, it may be difficult to confirm deception when an anonymous user baits or spams a virtual space (or is perhaps not even human) and when a user is authentic (Goldman-Hasbun, 2022). Goldman-Hasbun’s work makes clear, one cannot assume that anonymous posts are not spamming the board. At the same time, the use of real names also comes with its own risks, for example introducing an observer effect or putting the observer in jeopardy. This does not necessarily invalidate the data gathered in these spaces, it is merely one methodological consideration around a particular field of interaction and observation. Before getting in the field ethnographers should ask themselves what kinds of threats to data validity might exist in the virtual worlds they are entering.

Other virtual spaces may not put value in anonymity, such as those of social media influencers, where anonymity is not as often strived for. However, such spaces are also susceptible to fraud or deception, such as through fabrication of experience. There may also be meaningful differences between individuals’ virtual and non-virtual personas. Motivating factors and dynamics in virtual space vary, and there is, for example, a great deal of financial reward for those who are able to attract a following in social media. Sociologist Forrest Stuart conducted an ethnography which linked virtual and physical personas, comparing online personas of ‘micro-celebrity’ musicians who identified with and reproduced online representations of urban gangs with observations made in the physical:

“I came to realize that a responsible account [of the online personas] required directing even more attention to the agency and ingenuity of these young people as they try to cash in on their stigma. It also forced me to continually remind readers that these online performances are often just that—performances.” (Stuart 2020, p. 216)

Stuart found that oftentimes online personas were exaggerated, for example, with individuals claiming online to have engaged in much more violence than was actually the case, though both personas influenced the other. Sometimes, individuals would try to live up to or maintain online performances in physical spaces in some ways, such as impressing the romantic partners in their lives. This deception was both intentional (the research subjects knew they were not being truthful about the violent acts not committed) and reflective of meaningful social processes. Unlike Colleen discussed in the preceding section, who momentarily took on personal attributes in order to conform to a study’s expectations without any personal knowledge of the field of research, the subjects of Stuart’s research meaningfully engaged with and navigated between both personas and fields. Identifying this deception and triangulating the data was meaningful for Stuart’s analysis, and at that point the ‘deception’ itself became an object of study. Nonetheless, one might imagine a very different analysis and/or set of research questions if Stuart had studied only the online personas.

How does one identify intentional deception and its meaning for data validity while also acknowledging that research subjects often have complex motivations and views? If attempting to identify deceptive data or planning for the possibility of deceptive data, a researcher may ask themselves:

1. *Does the virtual space contain the ‘whole story’? If not, what ‘part’ does it reflect?* By studying one virtual space in which his subjects expressed themselves and comparing it to data gathered in-person, Stuart found that the virtual space contained an element of performance whereby subjects would enhance their accomplishments for the benefit of the virtual ‘audience’. Ascertaining which part of the ‘story’ that the virtual space contained, was key to Stuart’s interpretation of the data. For every online space with a public, accessible space, there will also be data which are harder to access, e.g., from private messages to proprietary algorithms. Obviously, this is analogous to in-person spaces--researchers are almost never privy to every private conversation or knowing glance– but it can help to think of all the ways one is not gathering data in a specific space.
2. *Many times, virtual spaces are created with specific intent, how does the intent shape the space and what occurs there?* For example, sociologist Robards (2017) studied a virtual forum (a subreddit) created for cismen identifying as straight to access gay porn and identity affirming interaction (2017). In the space, users and moderators encouraged non-judgmental discourse around fluid sexual identification. In other words, the space was created with a specific intent which was reflected in the way the space was maintained (and, then, constrained). One might imagine a very different discourse in another forum which was created with a different intent. Whereas analysis of either space could perhaps provide interesting insight into sexual identity or the ideologies around specific norms or practices, either space would also likely be limited in the types of research questions they might reasonably speak to, given each’s specificity and constraints.
3. *In what ways can the data be feasibly triangulated? Has this been planned for in the research design, questions and analysis?* Each study will likely have its own unique considerations around triangulation. A few methods of triangulation are discussed in the next section but for now the point is that it is at the phase of designing research that the ethnographer should think about how to go about collecting data such that data can be triangulated.
4. *What motivates, directly and indirectly, interaction in the virtual space?* For Colleen, money was a motivating factor. For many participating in social media spaces, influence, connection, popularity, and also income can be motivating factors. In such spaces, the norms of a virtual community can also motivate (or not) interaction in virtual space.
5. *Are you making any assumptions about data validity in the research design?* For example, there are inevitable risks in assuming that those anonymously posting on message boards are not merely spamming the board (or that they are not bots). For Goldman-Hasbun, assuming this risk and the validity of the data was central to the research questions they were able to explore. At the same time, there are also risks in relying on ‘real’ names and likenesses, as this does not preclude deception. Before getting into the field, ethnographers should ask themselves what kinds of threats to data validity might exist in the virtual worlds they are entering.
6. *Are there aspects of the research field which make it more or less vulnerable to deception?* In Forberg’s (2021) study, there was an interplay between a norm of anonymity and an expectation that the space could be infiltrated by imposters. Driving this, at least in part, was that the space was dominated by individuals with extreme and politicized ideologies, which was thought to risk attracting those who did not share extreme beliefs. A norm of anonymity both reflected an assessment of this risk (in Forberg’s (2021) words, that individuals using legal names could be doxxed), and a vulnerability to deceptive participants who for whatever reason might pretend to share the underlying beliefs of the space.

#### The data gathering & analysis^2^ phase: identifying deceptive data

Confirmation and triangulation of data can be accomplished in several ways, both while gathering data and in analyzing the data. Some sociologists have hired independent ‘fact checkers’ to double check their work, other methods of triangulation include verifying data independently, cross-checking multiple sources or mediums of data, verifying data across a number of interactions with the same subject, or cross-verifying data with different research subjects. Methods of triangulation will vary by study and possibly as the researcher learns more about the field. Colleen claimed to be a public housing resident, though she was not one. I set forth some guidelines based on the ways in which I identified Colleen as being deceptive about her qualifications and experience, as well as some methods for understanding deception which I adopted after my experience with Colleen.

1. *Compare data across research subjects.* I was able to understand that Colleen was most likely not who she said she was when I asked her standardized interview questions which she then answered implausibly. For example, I asked her how she acquired her public housing apartment. To this question she answered that she had filled out some paperwork and received the apartment within a few weeks. In comparison to the range of other stories I had heard--the apartment had been in the family for generations, they’d waited on lists for years and years, they’d come from a shelter--her answer was outlandish.
2. *Generate data to be triangulated independently.* After my experience with Colleen, I also further built in deception checks for interview participants, for example, asking them in synchronous conversations questions like “What is your favorite restaurant close to your home?” or “Where do you shop for groceries?” as this would produce data that could be easily triangulated by searching to see if that place indeed existed and was nearby a stated residence if a subject was able to produce a plausible, timely answer.
3. *Cross-verify stories.* After interviewing the ‘real’ Colleen, I became curious if the stories she was telling me were *really* real. I was able to cross check her stories with sources I found online in order to verify important parts of them. For example, I looked for anything related to her reported work as a university instructor, finding documents such as a press release about an award she had been given at the university she worked at, and a program for a conference.
4. *Require conversational synchronicity when possible.* Colleen was able to easily fabricate answers to questions that were not asked synchronously, in synchronous conversations she had more trouble.
5. *Take multiple observations.* It stands to reason that I would have been able to gather more insight into Colleen’s fabricated identity across multiple interviews or observations. In an online ethnography, observations across time that yield cohesive, if evolving, narratives can confirm data validity or facticity.
6. *Take note of outliers*. Taking notes or coding for extreme views may help an ethnographer identify deception. Many of the stories that Colleen told were outliers--they were extremely different from the stories I had heard before responding to similar questions. In my own virtual ethnography of adoptive parents, I observed for more than two years parents who actively participated in social media-based networks. These parents existed across a range of beliefs about adoption as a moral good (or evil). In my notes and analysis, I noted where parents held extreme views about adoption (i.e., rigid ideologies that adoption was ALWAYS evil or ALWAYS good), and focused my study primarily on parents who were somewhere in the middle of that ideological spectrum. I did not discard the ‘extreme’ data, but I chose not to focus on it, and noted when I used data from ‘extreme’ research subjects. In the community, where extremism was *not* the norm, provocateurs with extreme views could sometimes seem more like ‘trolls’. Whether or not the extreme subjects were deceptive, and I cannot say whether they are more likely to be deceptive or not, it did help me to make sense of my data to sort it that way.

I want to be clear that not every field of research would be able to accommodate these specific methods, and I am not suggesting one try to do so. Additionally, there are many traditional methods of verification of participant identity which require consideration according to individual research paradigms, for example asking for a legal ID. This type of identity verification might be important for some types of research, but could have negative effects in others. For example, in a study of public housing residents, a researcher might initially consider asking for a lease in order to verify identity. However, asking for a lease does not necessarily serve that purpose as thousands of ‘real’ public housing residents live in public housing off-lease. Asking for a lease might unnecessarily, and to the possible detriment of the study, overly-narrow a pool of research subjects. There is also the matter of rapport building--asking to see a lease also has the potential to alienate research subjects who are at times wary of revealing too much personal data online and could seem stilted when developing relationships with research subjects. Snowball sampling might also be a way to both verify identity and recruit research participants who are less likely to be deceptive if studying people who are part of specific communities. However, consider as well that research subjects might either be wary of identifying other individuals involved in sensitive communities or may pass on the contacts of other deceptive individuals.

#### The analysis & reporting stage: what to do with deceptive data

The issue of data validity and facticity is central for ethnographers, including practical matters around the handling of data and how to share invalid deceptive data with readers. So too, are questions around data transparency as an indicator of legitimacy and rigor in ethnographic study. Therefore ethnographers must consider what they will do with any deceptive data if collected when it comes to analysis and writing up of results.

Colleen was deceptive, though in my first interaction with her, I could not confirm that she was deceptive. Rather, I only could suspect she was because of the implausibility of her answers to my standardized questions. While the waste of time (and money, I did send her the promised incentive fee) was an annoyance, I did not accuse her of deception in our first interactions as I felt it would be unprofessional to do so. When my undergraduate Sociology students first read a draft of my article about Colleen, this is the thing that deeply troubled them. Shouldn’t I have confronted Colleen about her deception and let her know that what she was doing was immoral? Generally, I would strongly caution ethnographers to avoid emotional confrontations or unreflective accusations of deception in the field, as I imagine it would not be productive in most cases. If an ethnographer feels that an interaction or observation might be based in deception, it might be worth reflecting first on ‘why’ before making further decisions about how to deal with or further confirm deception.

Data transparency requires that ethnographers make transparent “how they know what they know” so that other scholars may judge for themselves the ethnographer’s claims (Murphy et al., 2021, p. 52). I suspect that many times, deception will be highly suspected but not absolutely confirmed. Even without confirmation, I felt that it would be wrong to include Colleen’s data in an analysis. I segregated her data, marking it as based on deception, and decided Colleen’s interview should not count toward my interview counts. Nevertheless, I also felt it was important to keep Colleen’s data and to deal with it as deceptive rather than pretending as if it did not exist (i.e., simply disposing of it). Simply burying deceptive data under the rug would not meet the ideals and requirements of data transparency. Neither would be the use of data without any thought to the possibilities of deception. Furthermore, how one handles and is transparent about deceptive data helps an ethnographer build trust with readers concerning their data more generally by giving the reader access into how thoughtful a researcher is around questions of data validity.

I’ve not come across many sociological studies that specifically discuss contact with intentionally deceptive participants, possibly because data that comes from interactions with those who intentionally deceive is thought to be not useful analytically. However, sociological studies do commonly note instances in which the accounts given by research participants vary from the researcher’s own experience. Data transparency requires that readers may analyze a researcher’s interpretations and consider other explanations as well as that researchers might ultimately return to data and re-assess it (Jerolmack, 2022). Ensuring transparency, while also ensuring data validity, might require the researcher to consider the following in terms of deceptive data when conducting data analysis:

1. *Reflect on whether suspected deceptive data can or should be further confirmed as deceptive.* When I first interviewed Colleen, I did not feel it was necessary to additionally confirm that her data was deceptive before treating it as deceptive because her answers to interview questions were such outliers.
2. *Segregate the deceptive data in analysis.* Instead of disposing of Colleen’s data, I clearly marked it and segregated it from other data.
3. *Retain your deceptive data as you do your non-deceptive data.* Deceptive data is still part of the dataset no matter how one treats it. Transparency does not necessarily require retention of data, though increasingly, data transparency requires that researchers not destroy their data (Murphy et al., 2021).

Additionally, researchers should also address deceptive data when writing results of research studies:

4. *Attend relevant considerations of the anonymization and redaction of deceptive data.* The ramifications of identification for deceptive participants may be different than those who are not deceptive. In a hyper-punitive ‘cancel’ culture, those identified as deceptive could be subject to far reaching and unpredictable consequences. This may be a consideration in studies where real names are used.
5. *Address deceptive data in methodological explanations sections of research articles, parentheticals or endnotes, including why it is suspected (or confirmed) as being deceptive or invalid.* This is one way to build trust with readers and aligns with the principles of data transparency. Giving your reader as much information as possible helps the reader evaluate your claims and to better understand ‘how you know what you know’. As well, disclosing such information may help your reader better understand the social context of your study and those in which you generalize findings. Understanding deception that occurs in one study also may help the reader to understand more about what conditions produce deception.

As well, researchers should not assume that all deceptive data is without value. Facticity is important in ethnography, though not always paramount, and at times accounts which cannot be corroborated can be valuable to research (Murphy et al., 2021). At various times, ethnographers have chosen to foreground their own observations over the accounts of the perceptions of others, and other sociologists have chosen to highlight the perceptions of their subjects, even when they conflict with their own (Murphy et al., 2021). Inevitably, decisions as to whether or not to include data in analysis, or whether to foreground it or not, will require application of the researcher’s judgment.

Ultimately, with Colleen, the decision to set her data to the side was a simple one--she was intentionally deceptive about the essential qualifications for the study, was motivated to participate by financial reasons, and had no personal experience with the field of study or anyone in it. However, without confirmation of this (only an understanding of her data as implausible) the initial decision to set her data aside was also a judgment call. The universe is eminently complex and a researcher cannot know everything. For example, one might imagine an alternate universe where Colleen *actually had* received her apartment after a few weeks of waiting. For example, off the cuff, she could have been illegally subletting and using a lightly invented cover story because she did not want to risk the illegality of her housing situation coming to light, or she could have been a criminal mastermind who engaged in identity theft to acquire the apartment, or a hacker who put herself at the top of the waiting list. Nonetheless, even considering a myriad of creative possibilities, I made a judgment that her data was, on the whole, implausible.

Of course, I eventually received an external ‘confirmation’ that Colleen was being deceptive and admission from Colleen herself that she was deceptive, but this is unlikely to be the case in most instances of deception. The principles of data transparency acknowledge the necessity of the exercise of judgment, while also safeguarding it by allowing others with different perspectives and bases of knowledge to assess whether the case is convincingly made (Murphy et al., 2021).

## Conclusion

While working through the peer review process originally while writing about Colleen and virtual interviewing (Owens, 2022), one reviewer was deeply concerned about how the presence of fraud or deception might seem to invalidate or discredit virtual interviewing as a methodology. I do not share this concern about virtual interviewing or other virtual methodologies. If anything, my experience with Colleen, anecdotally at least, shows that identifying and contextualizing deception in virtual research is readily possible and possibly valuable.

In this essay, I take the liberty of identifying prescriptive guidelines concerning deception which researchers might ask themselves or choose to implement when designing methodology for ethnography of virtual spaces. I want to reiterate the (perhaps) obvious that each virtual space will be different, and will present its own considerations, advantages and disadvantages for research and research questions. This essay is a provocation for research designers, and is not meant to indicate that certain virtual spaces are valuable or invaluable in terms of the data they may produce.

The various challenges of virtual ethnography, as well as its promise, are known. The centrality of the virtual in social life is clear, and Sociologists must include virtual spaces in their work to continue to shed relevant light on social processes. Virtual ethnography presents extraordinary possibilities, even while “methodological training [for virtual ethnography] is still in its infancy” (Urbanik and Roks, 2020, p. 213). Promisingly, the ‘natural experiment’ of ethnographic methodology in the years following the Covid-19 pandemic has been an excellent opportunity to understand more about the risks and methodological differences between in-person and virtual ethnographies. It is imperative for researchers involved in these studies to memorialize the methodological puzzles they encountered over the course of their research, including how they sought to confirm data. To do so will further establish the gold standard status of virtual ethnography in terms of methodological rigor as the discipline progresses further into the study of virtual social life.

## Data availability statement

The data analyzed in this study is subject to the following licenses/restrictions: confidentiality. Requests to access these datasets should be directed to lisalucile1@gmail.com.

## Ethics statement

The studies involving humans were approved by Institutional Review Board, Columbia University. The studies were conducted in accordance with the local legislation and institutional requirements. The participants provided their written informed consent to participate in this study. Written informed consent was obtained from the individual(s) for the publication of any potentially identifiable images or data included in this article.

## Author contributions

The author confirms being the sole contributor of this work and has approved it for publication.

## Footnotes

^1^Colleen’s Name has been changed. A longer form article written on Colleen is also available as an open source from Sociological Methodology at https://journals.sagepub.com/doi/full/10.1177/00811750221106777

^2^Both this section and the next discuss the “analysis” stage. This acknowledges that analysis happens both alongside data collection and prior and alongwith the writing process.
